# Household transitions between ages 5 and 15 and educational outcomes: Fathers and grandparents in Peru

**DOI:** 10.4054/demres.2022.46.14

**Published:** 2022-03-15

**Authors:** Sarah A. Reynolds

**Affiliations:** 1 University of California, Berkeley, USA

## Abstract

**BACKGROUND:**

Latin America has high rates of single motherhood and intergenerational coresidence, resulting in children experiencing changes in household composition – particularly with respect to fathers and grandparents. In other contexts, such changes have been shown to influence educational outcomes.

**OBJECTIVE:**

To test if the presence of grandparents and fathers in the household are differentially associated with educational outcomes during schooling years in Peru.

**METHODS:**

Young Lives longitudinal data consist of around 2,000 children who were followed from age 1 to age 15 between 2002 and 2017. Using value-added and child fixed effects models, I examine if the number of changes in household structure involving fathers and grandparents, the type of change (exit or entrance), and the identity of the household members are associated with cognitive outcomes. Persistence was tested as well as heterogeneous associations by child’s age at transition and disadvantage.

**RESULTS:**

More than half the children experienced a change in household composition between ages 5 and 15. Father separation was associated with worse cognitive scores and lower likelihood of being on-grade. This was strongest if separation occurred when children were older. Grandparent presence in the household was not as strongly correlated with child outcomes, but results suggest that children have better cognitive performance after grandparent separation from the household. Associations between household composition and child outcomes were stronger if children were disadvantaged.

**CONTRIBUTION:**

This research provides evidence that fathers and grandparents are both important contributors to child educational outcomes in a context where three-generational households are common.

## 1. Introduction

Children in Peru live in diverse family structures, with less than one-fifth of Peruvian children consistently living in nuclear families for their entire childhood. Specifically, coresidence with grandparents is common, with more than a third coresiding with grandparents at some point during childhood, and 40% experience father absence (Cakouros and Reynolds 2021). Similar to many Latin American countries, Peru has a high rate of adolescent pregnancy, with around 20% of females having their first child by age 19 (Favara, Lavado, and Sanchez 2016); many of these young women remain cohabitating with their parents for some time until they are ready to become independent. Recent research on Peru also finds that single-mother-headed households are on the rise (Cuesta, Rios-Salas, and Meyer 2017).

Given the variety of and movement between family structures, it is important to understand the influence of these household transitions on children’s outcomes. In particular, the experience of a household transition has been shown to be linked to child cognition and schooling attainment. The focus has typically been on fathers, as the bulk of the research on non-nuclear family structures has focused on father absence. Review articles find that, in the United States, father absence is negatively associated with children’s socio-emotional well-being, cognitive test scores, and academic achievement (Amato and Gilbreth 1999; McLanahan, Tach, and Schneider 2013). In the international context, where father separation has been most studied with relation to migration, there are mixed findings of associations between parental migration and children’s educational, health, and labor outcomes (Antman 2013). In Latin America, cross-sectional data indicate that children with coresiding fathers have better educational progress than children without coresiding fathers (DeRose et al. 2017).

Recent literature, however, indicates that the household transitions of nonparental family members can have associations with child education that are as large as the associations with parental transitions (Perkins 2019). Several studies found that child coresidence with grandparents is associated with better child outcomes in single-mother households (Aquilino 1996; Deleire and Kalil 2002; Dunifon and Kowaleski-Jones 2007; Monserud and Elder 2011), although Monserud and Elder did not find associations between grandparent presence and child educational attainment when both parents are in the household (2011). A study examining heterogeneity of effects of grandparents by household wealth found significant benefits of grandparent coresidence in terms of school readiness for children born to single mothers in richer households but no effects for children born to poorer single mothers (Augustine and Raley 2013). Research on Latin America indicates that a high fraction of children experience changes in grandparent coresidence (Cakouros and Reynolds 2021; Reynolds et al. 2018), but estimates of associations with child outcomes have been limited to early childhood in Chile (Reynolds et al. 2018) and health outcomes of child anemia in Mexico (Schmeer 2013).

Child cognitive achievement and schooling achievement are both important outcomes relating to adult economic well-being in Peru. In a study examining developing countries, private gains to completing an additional year of schooling are associated with a 7.6% increase in wages, and this is generally higher within Latin America (Peet, Fink, and Fawzi 2015). A study of urban Peru shows that an increase in workers’ overall cognitive scores by one standard deviation is associated with 9% higher earnings (Díaz, Arias, and Tudela 2012). Nevertheless, almost half of Peruvian ninth-graders finish that grade without achieving basic reading skills (Hanushek and Woessmann 2008). For both math and reading, Peru scores in the lowest 15% of countries that participated in the Program for International Student Assessment (PISA) (Vilena et al. 2017). The average math score of Peruvian 15-year-olds was at level 1, while the average score of students in Singapore, the country with the highest average, was level 4. (The maximum level is 6.) The gap was smaller for reading, with the average Peruvian reading score hovering between levels 1 and 2, and the highest average score (Singapore’s) was not yet at level 4. Furthermore, the scores of students in urban in contrast to rural schools and in public in contrast to private schools remain a full level apart for both math and reading. More than a fourth of children reported having repeated a grade on the 2009 PISA (Ikeda 2011). This results in costs to the education system and to the student, who may delay entry into the labor force or higher education or may even drop out of school.

In this paper, I examine associations between changes in household composition of fathers and grandparents with children’s vocabulary attainment, math skills, and being on-grade. Using value-added (lagged dependent) variable models, I test if the number and type of transitions (separation or union of fathers and grandparents) experienced between ages 5 and 15 are associated with age-standardized test scores and being on-grade in school. I find the number of household transitions to matter only for being on-grade in school, but father separation specifically is negatively associated with all outcomes; there are much smaller associations found with grandparent transitions and father union. A child fixed effects analysis finds a concurrent negative association between father absence and math scores, though this association weakens with time. Heterogeneity analysis along various measures of advantage suggests that, in the most vulnerable households, children’s vocabulary attainment, math skills, and being on-grade in school are negatively associated with father separation and that grandparent presence is associated with worse cognitive scores. These results indicate that educational support that responds to students’ household dynamics may be useful.

## 2. The demography of the Peruvian family

Peruvian extended families have been of interest to social scientists for more than 50 years. In 1961 Hammel categorized women in the semirural village San Juan Bautista based on their household structure: nuclear, nuclear extended by members of subsequent or previous generations, or unpartnered women who were dependents or nondependents (1961). Family structure patterns were not remarkably different from current patterns, with younger mothers more likely to coreside with their parents and transition to independence at later ages. Similarly, Tienda (1980) found that in Peru, single-parent families were about 1.5 times more likely to coreside with one or more nonnuclear relatives than two-parent families; single mothers could have been substituting the support of fathers with that of grandparents.

This pattern was not unique for the Latin American region; in Mexico and Colombia, most children living with unpartnered mothers also coresided with grandparents (Richter 1988). Fertility patterns in Peru have been similar to those of other Latin American countries: a large decline in the fertility rate (dropping from near 7 in 1960 to 2.3 in 2017) with a slower drop in the rate of adolescent motherhood (currently 58.8 births per 1,000 women ages 15–19) (World Bank 2019). Thus the current ratio of youths to elderly is smaller than it used to be, as is the case in countries in the fourth stage of the demographic transition. Grandparents may be more able to support children as they spread their wealth across fewer grandchildren than past generations did.

Recent economic factors have also influenced Peruvian families. Growth has been strong in the new millennium, but inequality persists; rural, subsistence farmers remain in extreme poverty, particularly among the indigenous population. There has been a great deal of migration toward the cities (Lees 2009), which has been accompanied by an increase in the female labor force participation rate (World Bank 2019). Working mothers have had to secure other sources of child care – relatives or centers. Furthermore, the increase in economic activity driven by extractive industries such as mining has made men’s short-term migration common. (Mining is seasonal due to technologies that require rainwater [Koster, Grote, and Winterhalder 2013].) These absences, as well as women’s new possibilities for economic autonomy, may partially explain why single-mother families increased between 2003 and 2012 in Peru (Cuesta, Rios-Salas, and Meyer 2017; Liu, Esteve, and Treviño 2017). On the other hand, in spite of a strong economy, prospects for youths are bleak: close to 34% of Peruvian youths find it difficult, or very difficult, to get by with their present household income. Youth unemployment remains high, with higher rates of unemployment among those with tertiary education (14.6%, compared to 8.7% for people with secondary education) (OECD Newsroom 2019). These factors may contribute to reliance on grandparents for economic support.

### 2.1 Theoretical framework

Humanistic theory provides a framework for understanding how household transitions may interrupt learning processes (Schunk 2012). The theory suggests that learning occurs best when other needs (material or psychological) are already met. Household transitions may make satisfying a child’s needs more or less challenging for a number of reasons. It has been well established that children in households with fathers have more income and economic resources (Amato 2010; McLanahan, Tach, and Schneider 2013; Page and Stevens 2004; Thomson, Hanson, and McLanahan 1994). Depending on the context, however, it is uncertain if grandparents increase household wealth. In economies where human capital accumulation over the life course results in higher wages over time or in economies where pensions are large, a grandparent presence in the household can improve the economic situation (Mutchler and Baker 2009; Minkler 1999). On the other hand, in economies where wages depend on physical labor, a grandparent may provide less income than other adults and may not be as large of a contributor as younger and more able-bodied family members (Ruggles and Heggeness 2008). At the extreme, frail coresiding grandparents may need care, which takes parental time away from children. As caregivers themselves, older grandparents with physical limitations may provide lower-quality care (Luo et al. 2012).

Psychological needs may also be influenced by a household transition. Research suggests that family instability causes stress for the child and may be the source of lower child development scores; the identity of the transitioning family member is not as important as the transition itself (Cavanagh and Huston 2006; Osborne and McLanahan 2007). The absence of a father can also cause a loss of social capital available to fathers but not mothers (such as more prestigious positions in business, local community governance, or religion) that could support child development by enhancing a child’s status or self-esteem (Astone et al. 1999; Pleck 2007). The absence of a grandparent may be less stigmatized than the separation of a father.

Vygotsky’s sociocultural learning theory suggests that students’ learning interacts with interpersonal factors for individual development (Schunk 2012). Thus a greater exposure to people who use more or more challenging vocabulary and more opportunities to use or observe mathematical problem solving can support learning. In particular, household members who have larger vocabularies and greater math ability can influence the children in the household. For example, Lugo-Gil and Tamis-LeMonda (2008) find positive reinforcement loops between parenting practices and children’s cognitive development at age 3 and prior. This relationship could extend further into childhood.

The developmental processes of learning in these different domains (vocabulary and math) could also influence the impact of interruptions from household transitions. Though children develop much of their vocabulary in early years (Gilkerson et al. 2018), oral vocabulary knowledge has been shown to grow into adolescence (ages 12–14) and is highly correlated with reading comprehension during these ages (Ricketts et al. 2020).^2^ Numeracy and mathematical skills also have important foundations in the early years (Aubrey, Godfrey, and Dahl 2006), but more complex problem solving, typically encountered as adolescents develop the capacity to hold in mind more multidimensional concepts and strategic thinking, requires additional access to inhibitory control and working memory (Dumontheil and Brookman-Byrne 2020). The development of these abilities plateaus much later, in the mid-20s, so there can be extensive growth in mathematics during this period. These two distinct trajectories suggest that transitions at older ages may influence mathematics skills more than vocabulary attainment.

There are also reasons to expect that changes in household composition would influence children’s grade attainment and ultimately secondary school completion, as grade repetition or over-age-for-grade has been associated with dropout in a variety of settings (Sunny et al. 2017; Jacob and Lefgren 2009; Cabrera-Hernandez 2021). First, noncognitive behavior may be more responsive than cognitive skills (Schweinhart et al. 2005). Family stress, which can be a result of household transitions, has been shown to be correlated negatively with academic attainment (Boynton-Jarrett, Hair, and Zuckerman 2013; Brotman et al. 2018). The outcome of on-grade at school incorporates noncognitive abilities, such as behavior and reliable attendance, in addition to reaching minimum academic standards to pass a grade.

### 2.2 Empirical evidence

#### Fathers and academic achievement.

A number of studies have shown that father absence is strongly associated with lower levels of academic achievement (especially high school graduation), but evidence is mixed with regard to test scores (McLanahan, Tach, and Schneider 2013). Specifically for vocabulary, Foster and Kalil (2007) find little association between family structure and vocabulary, although Cooper et. al. find that more transitions (both coresidence with and dating of mothers’ romantic partners, which can include biological fathers) are negatively associated with children’s verbal ability at age 5. Discrepancies in these results may arise from Foster and Kalil’s (2009) exploration of family types, such as extended and single-mother households, but they do not include transitions of romantic partners who do not core side. For example, a paper on Peruvian fathers indicates that their absence is associated with lower nutrition (Dearden et al. 2013).

#### Grandparents and academic achievement.

Several studies have indicated that grandparent coresidence benefits grandchildren’s learning (see Sadruddin et al. 2019 for a review). Using Taiwanese panel data, Pong and Chen (2010) found that long-term coresidence with grandparents is associated with higher cognitive test scores in young adolescents; a recent transition into coresidence confers no such advantage. In contrast, data from rural China suggest that living with grandparents of low levels of schooling compared to the general population does not affect grandchildren’s educational attainment, but living with relatively well-schooled grandparents is significantly associated with a lower likelihood of school dropout (Zeng and Xie 2014). In Brazil, grandchildren living with grandfathers had better literacy outcomes (Ponczek, 2011), and children living with grandparents had higher expenditures on schooling (Renteria Perez, Turra, and Lanza Queiroz 2006). There are exceptions to this pattern of positive associations between grandparent coresidence and child cognition, however, including a study from Peru: Cross-sectional data found that children aged 6 to 14 living with grandparents had lower schooling expenses than those not living with grandparents, controlling for a variety of demographic and socioeconomic-status variables (Renteria Perez, Turra, and Lanza Queiroz 2006). Additional evidence comes from OECD countries, where 15-year-olds without a coresident grandparent had higher science scores than 15-year-olds with a grandparent in the household (Chiu 2007).

#### Studies considering both fathers and grandparents and academic achievement.

One study on Latin America that included a cognitive outcome considered the coresidence of both grandparents and fathers. In Chile, grandparent coresidence supports young children’s vocabulary while father coresidence provides income support (Reynolds et al. 2018). In the United States, Monserud and Elder (2011) found that grandparent coresidence was not associated with grandchildren’s high school completion or college enrollment with a two-parent family but was beneficial for youths in single-mother households. The findings of these two studies suggest that grandparents and fathers provide distinct contributions to children’s learning and schooling.

### 2.3 Theory and evidence on related themes

#### Timing of transitions.

There is also a small body of literature focusing on father departure and the impact of the timing on children’s outcomes. Early childhood is key for secure attachment, which strengthens children’s ability to develop and maintain healthy social relationships (summarized by Bretherton 1985). In contrast – though similarly focused on these relationship outcomes – social cognitive theory indicates that children have the capacity to understand relationship dynamics only by middle childhood, such that earlier transitions may not be imbued with as much loss as those experienced later (Bandura 1986). Several studies from the United States found that instability during early childhood, prior to elementary school, resulted in more negative behaviors (Cavanagh and Huston 2006; Ryan and Claessens 2013; Ryan, Claessens, and Markowitz 2015), although data from the United Kingdom show that father departure in early childhood did not yield much concern while paternal departure between ages 7 and 14 was associated with an increase in behavior problems (Fitzsimons and Villadsen 2019). Mikulincer, Shaver, and Pereg (2003) indicate mechanisms through which attachment can influence cognitive outcomes. For example, nonsecurely attached children are more adverse to novel and uncertain information. However, the literature on the timing of family transitions and cognitive outcomes is slim. Aughinbaugh, Pierret, and Rothstein (2005) find little evidence of the impact of timing of parental separation on behavioral and cognitive scores of youths in the United States. Lansford et al. (2006) find that parental separation between kindergarten and grade five (approximately ages 5–10) is more negatively related to behavioral problems, but later separation between grades six and ten (approximately ages 11–15) is more negatively related to grades assigned by teachers.

#### Modification by advantage.

Context could moderate the associations between family structure and child cognitive outcomes. In rural areas, which are poorer in Peru (Andersen et al. 2015), children are more isolated; thus family transitions could be more strongly associated with child outcomes than in urban areas, where children interact with more people who influence their learning. In Ethiopia, families of urban children focus on schooling success while rural families focus on children’s contributions to the household (Kassa 2016). Thus, when a household loses a member, rural children may need to participate in farming instead of school.

Fathers’ education may also influence the association between family structure and child development. Fathers with fewer years of schooling may have more stress and thus be less able to interact with non-coresident children after a separation, resulting in a greater association between father absence and child outcomes among less educated fathers. Cooksey and Craig (1998) found that in the United States, more educated nonresident fathers are more likely to have contact with their children than less educated nonresident fathers. On the other hand, the absence of more educated fathers could mean a loss of interactions that are more stimulating cognitively. Research from the United Kingdom finds that children of more educated fathers have worse associations between divorce and cognition (Mandemakers and Kalmijn 2014). There could be parallel mechanisms with respect to grandparent education, since less educated women on average have more children (Tuman, Ayoub, and Roth-Johnson 2007). Thus the grandparents may spread their familial support more thinly, having less time to spend with non-coresident grandchildren.

### 2.4 Contributions and research questions

This research from Peru adds to the literature on household structure and child development by providing a contrasting economic and cultural context; most studies on family structure and child development focus on upper-income countries (Perkins 2019). The inclusion of grandparents is reflective of changes in household structure experienced by children in societies with high rates of intergenerational coresidence. Even within Peru and Latin America, most previous research on household structure and child development focuses on father separation and child well-being (e.g., Dearden et al. 2013; DeRose et al. 2017). Yet data indicate that in several lower- and middle-income contexts, including Peru, a separation of the child’s household from the grandparents’ household is very common (Cakouros and Reynolds 2021). Some research on Latin America has compared the well-being of mothers who reside with male partners to the well-being of mothers who reside with their parents (Liu, Esteve, and Treviño 2017; Chant 2003), but only a few studies addresses how both father and grandparent presence may be differentially associated with child outcomes (Schmeer 2013; Reynolds et al. 2018). The current study on Peru adds to this small body of evidence with a primary research question: Are there associations between household composition – considering both father and grandparent presence as well as the direction of transitions – and children’s vocabulary attainment, mathematics skill, and on-grade outcomes?

The current study covers ten years of a child’s school years with four survey rounds. The previous highlighted studies on fathers and grandparents (Schmeer 2013; Reynolds et al. 2018) examined differences over only two or three years using two rounds of data. The multiple time periods spanning a broader range of school years allow for two additional research questions that take advantage of the temporal aspect of the data: Do associations between family structure and child outcomes persist over time? Is there evidence of a period of sensitivity to family structure in that there are differential strengths in the associations between family structure and child outcomes at different ages? The answers to these questions are important for determining if and when interventions to help children adjust to changes in family structure could be useful.

Finally, stratification analyses contrast the associations between household transitions and child cognitive outcomes for children in disadvantaged households and children in more advantaged households. The urban–rural stratification provides insight as to how the role of family members may differ with population density, and the stratification by father and grandparent education suggests mechanisms behind how family members influence children’s cognitive development.

This study on Peru concludes that father separation is associated with worse cognitive scores and a lower likelihood of being on-grade. Grandparent presence in the household is not as strongly correlated with child outcomes, but results suggest that children have better cognitive performance after grandparent separation from the household. The associations between household composition and child outcomes are stronger if children are disadvantaged, indicating that policy interventions around changes in household structure may be useful for children in rural areas or with less educated fathers.

## 3. Data

Data are from the Young Lives study from Peru (Barnett et al. 2013). A total of 2,052 children were first surveyed in 2002, and 1,813 children (88%) were present in all rounds. The children had a mean age of 11.7 months in 2002, 5.3 years in 2006, 7.9 years in 2009, 12.0 years in 2012, and 14.9 years in 2017. In this paper, the rounds are referred to by the ages 1 year, 5 years, 8 years, 12 years, and 15 years. The Central University Research Ethics Committee of the University of Oxford and the Instituto de Investigación Nutricional in Peru reviewed Young Lives survey protocol. Consent was obtained collectively from communities and individually from caregivers, and from children when they were old enough to provide it.

Participants were selected using a multistage, cluster-stratified, random sampling process that excluded the richest 5% of districts (Brock 2011). At the first stage, ten random draws of 20 sites were made. The composition of each draw was rated for accessibility and diversity to ensure it reflected the Young Lives study aims of examining the causes and consequences of childhood poverty and the diversity of childhood experiences. Within the sentinel sites, approximately 100 children within the eligible age category were randomly sampled for participation; there was only one study child per household. Less than 2% of selected households refused to participate. Additional details regarding country-specific sampling protocols and strategies can be found in country reports accessible at www.younglives.org.uk.

### 3.1 Outcome variables

The Peabody Picture Vocabulary Test assesses language development. It has been translated into Spanish and validated in Mexico and Puerto Rico (Dunn and Dunn 1997). Children were tested with the Spanish version of the test (whose Spanish acronym is TVIP) (Dunn et al. 1986) at ages 5, 8, 12, and 15. The adapters of the Spanish test used item analyses to determine which words to include; some items differed from those on the English version. The TVIP has been widely used throughout Latin America and appears to be effective in detecting the impact of various interventions on language development (Crookston et al. 2011). Scores from the TVIP administered in an indigenous language (N = 217 at age 5, N = 204 at age 8, N = 63 at age 12, and N = 39 at age 15) were excluded from the analysis due to incomparability. Round-specific details about the test, including selection of questions and implementation, can be found elsewhere (Cueto et al. 2009; Cueto and Leon 2012).

Children were also tested in math at ages 5, 8, 12, and 15. The test at age 5 differed from the tests at later ages in that it was the quantity subtest of the Cognitive Development Assessment (Cueto and Leon 2012). It was substituted in later years with existing items from national and international testing programs that had been published freely, and testers developed a few new items based on measures that were commonly used to assess mathematics skills because the original test would have been too easy for most children, resulting in ceiling effects. The first section aimed to measure basic quantitative and number notions. It included nine items on counting, knowledge of numbers, number discrimination, and using basic operations. These questions were read by the fieldworker with the aid of cards, so that no interference would result from poor reading skills. The second section included 20 word problems with whole numbers. The items were ordered in increasing levels of difficulty (according to the pilot test). Each child took the test at his or her own pace, but the second portion of the test was gently discontinued after eight minutes at age 8 and ten minutes at older ages. At ages 12 and 15, the first section was fully computational using basic operations, and the latter section included problem solving questions; these were released items that were publicly available from Trends in International Mathematics and Science Survey (TIMSS) and PISA. These topics included (1) data interpretation, (2) number problem solving, (3) measurement, and (4) basic knowledge of geometry. Further information on the psychometric characteristics of these tests has been documented elsewhere (Cueto and Leon 2012).

**Figure 1:**
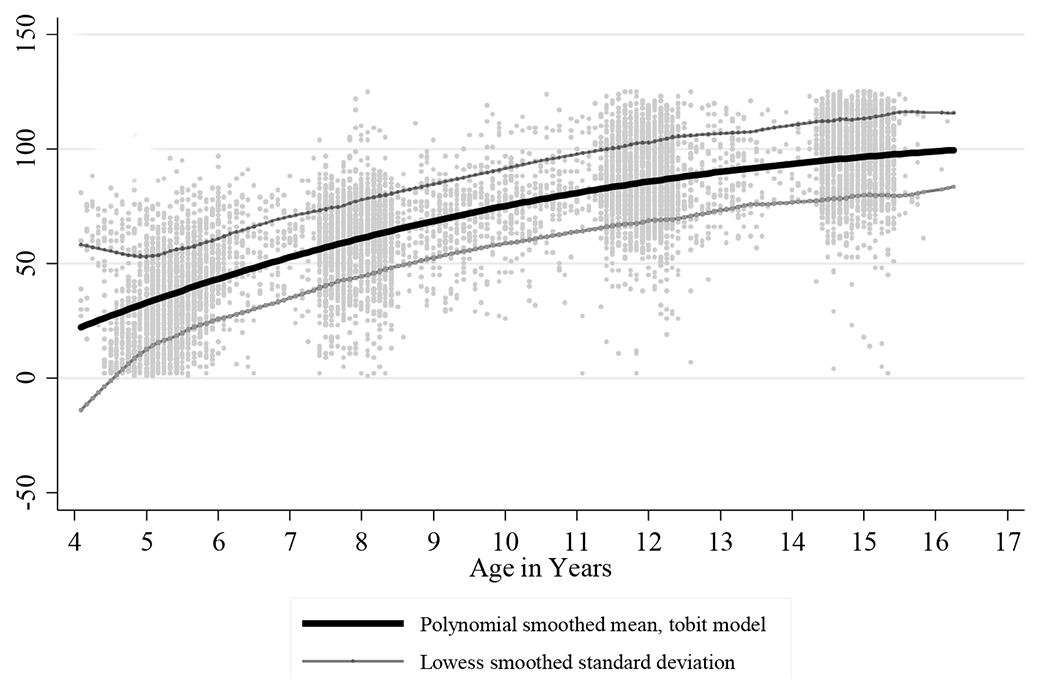
Fitted mean and standard deviation used in standardization of TVIP by age *Source*: Young Lives Peru. *Notes:* Data clumps are focus children tested around ages 5, 8, 12, and 15. Interior scatters are younger siblings surveyed when the focus children were ages 8, 12, and 15.

**Figure 2:**
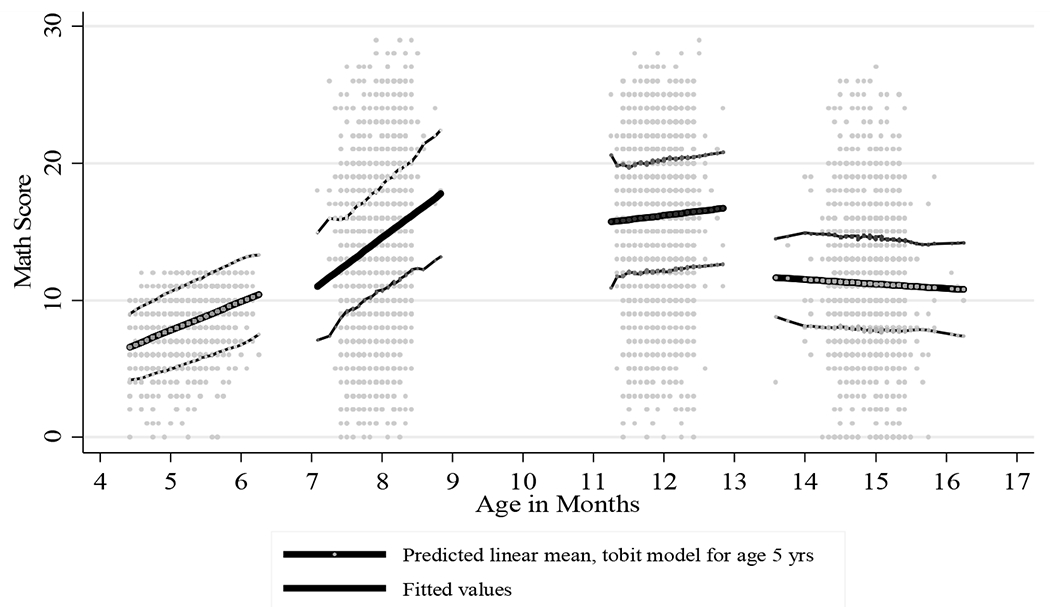
Fitted mean and standard deviation used in standardization of math skills by age *Source*: Young Lives Peru. *Notes:* Focus children were tested around ages 5, 8, 12, and 15. Tobit adjustment at age 5 accounts for ceiling effects. These age standardizations were used in the analysis with the exception of that for age 15; the full group was standardized together instead of by age.

Age-standardized scores were used to compare results of the cognitive tests over time. Younger siblings were also given the TVIP when the focal child was age 8, 12, and 15, providing more data points for the standardization. All observations were used to standardize math outcomes, but vocabulary outcomes were not used for children younger than age 4.5 (N = 82). The omitted data primarily came from younger siblings and were sparser across age. The means and standard deviations used to calculate the age-and language-standardized TVIP scores were generated by applying a methodology similar to that used by Rubio-Codina et al. (2015), though it was adapted using a tobit model of a third-degree polynomial to take into account floor effects; many of the youngest children scored 0. The age-specific means of the math scores were generated by a linear fit within each round of data, which was confirmed as the best model when testing multivariable fractional polynomial options with the Stata command mfp. Tobit adjustment was used for the first-round math assessment to take into account floor effects. For both the vocabulary and math assessments, the age-conditional standard deviation was calculated by squaring the residuals of the function, and the Lowess smoothing method was used to generate age-conditional standard deviations (bandwidth 0.1). Standardized values beyond four standard deviations were replaced with 4 and −4 (TVIP N = 3, math N = 5). The smoothing functions of the age-specific means for both cognitive assessments are illustrated in Figures 1 and 2.

An exception to age-specific means and standard deviations was math scores at age 15, where the linear fit had a negative slope (Figure 2). Since there is no theoretical basis to suggest this relationship,^3^ the overall mean and standard deviation were used for the last round’s standardized math scores rather than adjusting these for age.

The binary outcome of on-grade in school was determined by adding the grade in school to the age at which the child started first grade. If this sum was less than the child’s age by two years or more, the child was considered delayed.

### 3.2 Household transition variables

First, household composition was determined at each round. An indicator variable was created for the type of family structure, indicated by the presence of different family members on the household roster in each survey round. *Nuclear families* had a biological mother and biological father present with no other adult coresiding. *Both parents* + *grandparent(s)* included a mother, father, and at least one grandparent. *Mother* + *grandparent(s)* families were households in which the child’s biological father was not present but at least one grandparent was. Children with a biological mother but without a biological father or grandparent were considered to be in *single-mother* families.

Grandmothers and grandfathers were not differentiated for this study because, using the child fixed effects analysis described in the estimation strategies section, coefficients for maternal grandparents were compared to those for paternal grandparents and those for grandmothers were compared to those for grandfathers. Family types were grouped based on “any grandparent present” (Table A-4).

A few other types of families beyond those defined just by father and grandparent presence were included as their own categories. The analysis does not focus on them because the sample size of these families is small (Table A-1). *Stepfather* families included a stepfather of the child. Stepfather families with grandparents were included in this category due to the small number of observations (N = 26). Extended two-parent families that did not include grandparents were categorized as *both parents* + *other adult*. Families of children who did not have a biological mother in the household were coded *no mother*. In all cases, families could include non-sibling or non-cousin adults (for example, an uncle or aunt) other than those indicated, with the exception of nuclear families. Most cousins are under age 18; their presence is taken into account with the control variables for other children in the household. Table A-1 shows, by family type, the portion of families with different relatives in the household.

Using these household structure categories, the number of transitions was determined by the number of changes between ages 5 and 15, with a maximum of three. For the four variables indicating father or grandparent separation and union, the presence of the specific household member (father or grandparent) was determined at age 5. If the household member was present at age 5 but was not present at one of the other survey rounds, this was categorized as a separation. If the household member was absent at age 5 but appeared in a later survey round, this was categorized as a union.

In most cases, a family member’s absence from the household was permanent. There were few cases of temporary absences for other reasons, so these were not distinguished from other types of absences. For example, at age 12, only 11% of fathers and 13% of mothers who had left the household were reported as being temporarily absent. However, this information was not available at all rounds, so for concordance with the other survey rounds, these temporarily absent individuals were not counted as household members. Similarly, there were few members exiting households due to death. For example, of the household members on rosters in the third survey round who were not present in the fourth one, only 6% were deceased.

### 3.3 Control variables

Child covariates were sex, firstborn, and school enrollment dummies. Except for the mother being indigenous (indicated by her native language being indigenous), parent covariates were continuous: years of education of mother, years of education of father, and age of mother at child age 5 years. SES covariates at age 5 were the three indices provided by the Young Lives survey: housing quality (averages of a measure of crowding between 0–1 for sleeping rooms per person and three dummy variables for the quality standards of wall, roofing, and flooring materials), consumer durables (fraction of the following items owned: a radio, television, bicycle, motorbike, automobile, landline phone, mobile phone, refrigerator, stove, blender, iron, and record player), and services (average of dummy variables for electricity, safe drinking water, sanitation, and adequate cooking fuel) (Azubuike and Briones 2016). The number of household moves was determined by the number of times the child’s household changed its community location, since within-community moves would be unlikely to interrupt schooling. This is an important control variable to include since school transitions could occur at the same time household separations occur.

Only time-variant control variables were needed in the child fixed effects specifications. Dummy variables for each round, child age in months, and their interactions were included.^4^ Also included was a variable to indicate if the child had moved communities since the previous round. To account for other children in the household, a set of variables indicating the number of children in that category (children 6 and under, boys 7–15 years old, and girls 7–15 years old), not including the child of interest, was included. The three indices that are components of the wealth index (housing quality, service access, and consumer durables) are included except when the outcome is the wealth index.

### 3.4 Stratification variables

The analyses were performed for different subsets of the population to see if transitions influence children differently across advantage. Separately examined were urban and rural children, children of fathers with secondary education or more and children of fathers with primary education or less, and children where the highest education level of any grandparent was secondary or more and children where the highest education level of any grandparent was primary or less. The grandparent education comparison was done for a smaller sample of children because information on grandparents is available only for children who ever had a grandparent living in the household.

### 3.5 Estimation strategies

I use a value-added model to test if the number of transitions matters and if the direction (separation or union) of father and grandparent transition influences child development during the school years. This is tested by examining if transitions are associated with worse child outcomes at age 15, controlling for the outcome at age 5. To test that greater instability leads to worse outcomes, the equation estimated is

(1) $${\text{Y}}_{15\;\text{k},\text{i}}=\beta {\text{N}}_{5,\text{i}}+\alpha {\text{M}}_{5,\text{i}}+\gamma {\text{X}}_{5,\text{i}}+{\text{Y}}_{5,\text{i}}+\varepsilon$$

$Y_{12k,i}$ is outcome k for child i at age 15. N_5, i_ is the number of changes in family structure between ages 5 and 15. M_5, i_ is an important control variable: the number of times the child changed communities between ages 5 and 15. X_5, i_ is a vector of control variables from age 5, including family structure indicator variables. Y_5, i_ is the lagged outcome variable from round 5. The error term is ε. The household structure from age 1 is not included in the transitions because the lagged cognitive scores from age 5 already incorporate the influence of household structure in that prior period. This value-added strategy helps control for factors such as a child’s indigenous language. Though the child may score low due to less exposure to Spanish, that would be reflected at both time points; the model measures a change in location in the age-standardized distribution over time.

A similar analysis is done for testing the direction of the transition for the separation and union of fathers and grandparents. (The transitions of household members with less movement/presence – such as mothers and stepfathers – are not explored.) The equation estimated is

(2) $${\text{Y}}_{15\;\text{k},\text{i}}=\beta {\text{T}}_{5,\text{i}}+\alpha {\text{M}}_{5,\text{i}}+\gamma {\text{X}}_{5,\text{i}}+{\text{Y}}_{5,\text{i}}+\varepsilon$$

which is very similar to equation (1) but which substitutes T_5, i_, a dummy variable for the transition under consideration, for N_5, i_. Additionally, a dummy variable for the presence of the other household member (father or grandparent) is included in X_5, i_. The error term is e. Differential influences of family type at different ages were tested by disaggregating the transitions to be changes between rounds at ages 5 and 8, ages 8 and 12, and ages 12 and 15. Due to the exploration of the direction, these analyses are limited to a subsample of families for whom the transition is feasible. For example, the sample for father separation is restricted to families in which a father is present in the household at first measurement.

The child fixed effects approach complements these analyses by allowing for the full sample to be analyzed in the same regression, taking into account all rounds of data and better accounting for selection bias by controlling for time-invariant factors. Outcomes of the same child are compared at different time points, at which the child has different family structures; the different family structures imply that a transition has occurred. Children without transitions do not contribute to the estimates of the importance of household structure, though they do contribute to the precision of estimating how the control variables associate with the outcome.

The equation estimated is

(3) $${\text{Y}}_{\text{T}\;\text{k},\text{i}}=\alpha {\text{F}}_{\text{T}\;\text{i}}+\gamma {\text{X}}_{\text{T}\;\text{i}}+\sum_{\text{i}}\tau_{i}{\text{FE}}_{\text{i}}+\text{T}+{\text{T}}_{\text{a}}+\mu$$

${\text{Y}}_{\text{T k},\text{i}}$ represents one of two outcomes k for child i from round T: age-standardized vocabulary score and age-standardized math score. (The on-grade outcome is not examined with this estimation strategy because on-grade is not recoverable.) F_T i_ is a vector of dummy variables indicating family structure of child i’s household in round T, with the omitted case being the nuclear family. X_T i_ is a vector time varying controls, including age in months. $\sum_{\text{i}}\tau_{\text{i}}{\text{FE}}_{\text{i}}$ is child fixed effects. T is a set of indicator variables for the survey round, which was also interacted with age T_a_.^5^ Standard errors are clustered at the child level; the error term is μ.

Two tests examined the temporal aspect of the associations between family structure and child outcomes. First, for both the directionality and child fixed effects analyses, differences in the main associations by age were examined. In the value-added approach, the separation was interacted with the age at which it occurred. (The experiences of union were too few to precisely estimate if transitions at different ages were differentially associated with child outcomes.) In the child fixed effects specification, differential associations of family type at different ages were also tested by adding interaction terms between the family structure indicator variable and the survey round indicator variables. Additionally, in the child fixed effects specification, a lagged family structure variable was examined to test for persistence of the associations of family structure with child outcomes. A smaller coefficient on the lagged value than on the current value would suggest fade-out impacts of transitions.

Finally, the data allow for a sensitivity analysis for the main child fixed effects specification using the vocabulary outcome, for which the outcome value was also available for younger siblings. This regression included observations of the siblings in addition to those of the focus child. Standard errors were clustered by household.

### 3.6 Sample selection

For the instability and direction of transitions analyses using the value-added model, the sample included focus children who had outcome data at ages 5 and 15. For the child fixed effects model, all children with at least two rounds of outcome data between age 5 and age 15 were included. The sample sizes were different for each outcome because not all children completed the cognitive tests and some children were not tested in Spanish for the TVIP. Data on household structure of the younger siblings were used in a sensitivity analysis for the TVIP outcome that included observations from younger siblings who had been tested in two rounds.^6^ Although the relation questions on the household roster referenced the focus child, the household structure of half-siblings could be different if a younger sibling’s biological father was the focus child’s stepfather. In such cases, adjustments were made accordingly: The younger sibling would be in a nuclear family while the focus child would be in a stepfather family.

## 4. Results

### 4.1 Descriptive results

More than half of children (57%) experienced a change in household structure at some point between ages 5 and 15 (Table 1). Of the children in all four survey rounds, the mean number of transitions was 0.7 across the four survey rounds; 33% had one transition, 14% had two transitions, and only 3% experienced the maximum of three transitions. A higher percentage of children experienced separation from a grandparent (20%) than separation from a father (18%). Grandparent union was also experienced by more children (13%) than father union (7%). The children experiencing grandparent separation were much more likely to be firstborn children, indicating that this dynamic was one of first-time mothers living with their parents and later becoming independent. Children who had experienced grandparent transitions were also more likely to have mothers whose native tongue was indigenous. Though the indigenous population is generally poorer in Peru, the SES variables were not lower for households of children experiencing grandparent separation, with the exception of the housing quality variable. This variable includes a measure of crowding, which likely drives this difference.

### 4.2 Main results: Are child outcomes associated with household structure?

#### Is the number of changes in household structure associated with child outcomes?

Results from the instability analysis suggest that the number of transitions in and of itself was not significantly associated with child cognitive outcomes but did reduce the likelihood that the child was on-grade in school by 3% (Table 2). The number of times the child changed communities, however, was positively associated with vocabulary attainment. This is likely because moves often occurred from rural to urban locations, which have more resourced schools and higher populations, providing more opportunities for conversational interaction. The age 5 cognitive scores for vocabulary and math were also strongly associated with the age 15 outcomes. A robustness check confirmed the linear model for number of transitions for on-grade but yielded conflicting results for the vocabulary outcome (results from the math outcome remained null): One transition was negatively associated with vocabulary attainment by 0.1 standard deviations (s.e. 0.04), but three transitions increased vocabulary attainment by 0.13 (s.e. 0.1). A further robustness check separating out the number of father transitions and the number of grandparent transitions indicated that father transitions were negatively associated with all outcomes (most strongly on-grade, with a coefficient of −0.08, s.e. 0.02), while grandparent transitions were positively associated with all outcomes (most strongly vocabulary, with a coefficient of 0.06, s.e. 0.03).

#### Is the direction (union/separation) of a household transition associated with child outcomes?

The analysis of identity and direction of transition further explores these differences (Table 3). The most consistent result was that father separation was negatively associated with all outcomes: Vocabulary and math were both 0.12 standard deviations lower (both with s.e. 0.06) among children who experienced father separation compared to children with both mother and father in the home; these children were also 11% less likely to be on-grade. Results for the other types of transitions were less strong, but it is notable that father union and grandparent separation had positive associations with the outcome variables (with the exception of father union and vocabulary attainment) and that grandparent union had negative associations with the outcome variables.

#### Are child outcomes associated with household structure within a child fixed effects framework?

Unlike the direction of transition analysis, which restricted the sample based on family structure at age 5, the child fixed effects analysis allows for an examination of all data points across various family types and provides estimates of the associations of family structure with cognitive outcomes among children who have experienced the transition. The type of transition is not specified in this model, but the family types indicate the presence or absence of different family members, providing additional information about the importance of fathers and grandparents in the household. No strong association between vocabulary and family structure is found (even with controls, considering lags, and considering a larger sample, including siblings), but there is one found between family structure and math scores (Table 4). Children in both family types without fathers present (the *mother* + *grandparent(s)* and *single-mother* families) have lower math scores (−0.15, s.e. 0.09, and −0.13, s.e. 0.07, standard deviations, respectively) compared to when they were living in a nuclear family.

### 4.3 Temporal analysis: Do the associations between family structure and child outcomes change with time?

The experiences of union were too few to precisely estimate whether transitions at different ages were differentially associated with child outcomes. For separation, this was tested with an interaction of the separation event with the child’s age at which it occurred. (Full tables are available upon request.) The negative association with cognitive outcomes was strongest for father separation between ages 12 and 15 (vocabulary −0.21, s.e. 0.1, and math −0.23, s.e. 0.1, standard deviations). Associations between outcomes and father union were of similar magnitude but slightly smaller. Associations were negative for transitions at younger ages, but their magnitude was less than 0.1 standard deviations. The negative association of father separation with on-grade was strongest at the youngest and oldest ages (17%, s.e. 0.05, and 12%, s.e. 0.04, respectively). The father separation association with on-grade at the middle age (8 to 12) was weakest (6%, s.e. 0.04). For grandparent separation, the association was not as precise or as large, but younger ages had stronger associations between grandparent separation and positive outcomes than ages 12–15.

However, these associations weaken with the lagged specification, suggesting that the negative associations between father absence and math scores may be temporary (Table 4). On the other hand, the coefficient on the *both parents* + *grandparent(s)* family type becomes stronger and more negative (−0.12 standard deviations, s.e. 0.06) under the lagged specification, suggesting that children experience accruing challenges even after grandparent coresidence.

### 4.4 Do the associations between family structure and child outcomes differ by advantage?

Stratification was examined along two lines of advantage: location (urban/rural) and education, considering both the father’s and grandparent’s education. Because the instability analysis did not yield associations, the stratification analysis was not applied. For the direction analysis, the results of the stratification analysis are presented only for father separation, for which, in some cases, sizable differences were found by advantage (Table 5). Differences in outcomes with respect to advantage types were small for the other types of transitions (such as father union and grandparent separation). For father separation, the associations were generally larger among disadvantaged populations. Most strikingly, however, if the grandparent had secondary education, father absence was not negatively associated with child cognition, and the negative association with on-grade was small (5% lower likelihood).

The contrast across disadvantage for child fixed effects analysis was not as strong (Table 6). However, some of the same patterns emerged. Among the children with advantage, there was generally a smaller association between household structure and cognitive outcomes than among the children experiencing disadvantage. Among children with a grandparent who had primary education or less, grandparent coresidence with the two parents resulted in lower scores, particularly in math, compared to when a child lived with two parents. This model controls for wealth index variables, so these differences are beyond those associated with economic constraints that may result in families coresiding. Interestingly, the presence of a grandparent with two parents was associated positively with vocabulary and math scores among rural families and in families where the father had primary education. However, among the disadvantaged, when the father was absent, the presence of a grandparent in the household with the mother was frequently associated with worse cognitive outcomes than if there was only a single mother.

## 5. Discussion

Children in Peru experience a large variety of household transitions during their school-age years (ages 5–15), with less than half of the children maintaining a stable family structure throughout this period. Grandparents are an important component in contributing to the variety of transitions, with 32% of children experiencing a change in grandparent presence in the household. Nevertheless, father separation is the primary transition negatively associated with the strongest change in cognitive scores and the likelihood of being on-grade in school. This finding is similar to those of studies from more developed countries (and thus primarily urban locations), which indicate that father absence is associated with worse academic outcomes (McLanahan, Tach, and Schneider 2013).

This study also provides suggestive evidence that grandparent transitions influence children’s educational outcomes: Children have higher scores after a grandparent transitions out of the household. These findings are most strong for children in disadvantaged households. Previous literature on grandparent coresidence in Latin America finds positive associations between grandparent coresidence and child development (Reynolds et al. 2018; Ponczek 2011; Renteria Perez, Turra, and Lanza Queiroz 2006), although previous cross-sectional research from Peru finds a negative association between grandparent coresidence and schooling expenses and a measure of on-grade for age (Renteria Perez, Turra, and Lanza Queiroz 2006). The study by Renteria Perez, Turra, and Lanza Queiroz (2006) explains the contrasting findings with Brazil, with the larger portion of intergenerational coresidence in Peru occurring among more impoverished grandparents who do not receive pensions. Thus grandparents may be competing for resources with their coresident grandchildren. This explanation resonates with the findings of this study, which also finds evidence that children who coreside with grandparents have worse academic outcomes, though in both studies, wealth is a control.

The longitudinal data reduce some concerns about selection bias, since controlling for cognitive scores at age 5 is done in the value-added model, and the child fixed effects model controls for time-invariant factors. There were some differences in results using the different estimation strategies. The findings using the child fixed effects model were smaller than those with the value-added model; in particular, the main analysis (child fixed effects) did not find associations between family structure and the vocabulary outcome. This distinction is consistent with the plateauing of vocabulary in later years, since the child fixed effects model measures deviation from an average standardized score over time while the value-added model better approximates a difference over the entire time period, which may be expected to be larger.

Findings that examined associations by age suggested that children may be more negatively influenced by father separation at older years than at younger years. This finding is aligned with findings from two studies that indicate that father separation in later years is associated with worse mental health (Fitzsimons and Villadsen 2019) and worse grades assigned by teachers (Lansford et al. 2006). One possible explanation of these findings comes from social cognitive theory (Bandura 1986): Older children better understand the relationship implications of loss behind father absence and thus are more impacted than younger children. This may be particularly the case in the machismo culture of Peru, in which a male presence in the household garners more esteem (DeRose, Huarcaya, and Salazar-Arango 2018).

On the other hand, findings that examined associations by age suggest that children may benefit more from grandparent separation at younger years than at older years. This finding has implications for vocabulary development, since early childhood is when the most growth in vocabulary occurs. Many older Peruvians still speak indigenous languages, so it is difficult to determine if grandparent hindrance of Spanish (the language of the test) vocabulary skills is due to use of the indigenous tongue in the household, lower levels of education leading to a less rich vocabulary during the interaction, or less educated grandparents interacting less verbally with the child. However, the finding that children of single mothers coresiding with grandparents who have primary education or less is associated with lower math scores than scores for children coresiding with single mothers without any grandparent present suggests challenges beyond language differences among some segments of the population.

Larger associations between father absence and child cognition are found among the most disadvantaged. Only 36% of Peruvian children with absent fathers receive child support payments (Cuesta, Hakovirta, and Jokela 2018); strengthening enforcement of child support payments could help ameliorate reduced wealth that may be associated with father absence and could even ameliorate relational ties, as fathers who pay child support stay involved with their children more than fathers who do not (Argys and Peters 2003; Weiss and Willis 1985). Certainly, in rural areas, children would be more isolated and thus family transitions would be more associated with child outcomes, as these relationships are the ones children depend on most for learning. In particular, the on-grade urban–rural gap associated with father separation is large, perhaps because in rural areas, children must take on more farming duties if a father is not present.

The non-null associations between father coresidence and child academic outcomes in this study contrast with evidence from developed nations that shows a weak association between father absence and child cognition (McLanahan, Tach, and Schneider 2013). The magnitudes of the associations in Peru are non-negligible in contrast to magnitudes found in other studies of policies that have been been shown to have associations with child development. Average effect size of 82 US preschool programs on cognitive and achievement scores was 0.35 standard deviations, though this drops to 0.21 standard deviations when weighted by the inverse of the squared standard errors to account for smaller programs having the highest effects (Duncan and Magnuson 2013). The average effect size of early child development programs in developing countries is larger, around 0.65. However effects are heterogeneous, with countries with better health outcomes (such as Peru) having smaller effect sizes (Rao et al. 2017). Most significant estimates in this study on Peruvian family structure were at least as large as the US estimates, with several larger than the developing country estimates, suggesting that policy around family structure has the potential to be as important for cognition as preschool programs.

There are several limitations to this study resulting from data limitations. Due to a lack of international or national standards for the vocabulary and math tests, the child development outcome variables are standardized to those of their peers; results should be interpreted as relative changes rather than absolute changes. There is low representativeness of some of the family members, so they are not richly characterized. Thus conclusions cannot be made about stepfathers, other adult family members besides grandparents, or mother transitions. Additionally, the survey lacks information on the exact timing of changes in household membership. In addition to family structure, future research should examine family functioning, which has been found to be a stronger predictor of math skills than family structure (Lin et al. 2019). However, this current line of research can be further developed to consider grandparents, a conderation that was not a component of the family functioning analysis. Similarly, indicators on relationship quality and (grand)parenting practices could be included in future studies. Although these are touched on for fathers in the survey, they are absent for grandparents, especially non-coresident grandparents who may still participate in childrearing.

The estimation strategy also has limitations. Although this longitudinal study reduces concern about selection bias by using lagged dependent variables and child fixed effects, all time-varying unobserved factors cannot be fully accounted for, so the conclusions cannot be claimed causal. The within-child comparisons remove from the estimates factors that do not change over time (for example, a mother’s birth order, nutritional status, or history of growing up in a two-parent family may influence her own coresidence with her parents), therefore greatly reducing concern about selection bias contaminating the estimates. However, unobserved time-varying unobservables, such as a dynamic genetic–environmental interaction in child development, could still play a role. For example, a learning disorder that worsens due to a mother’s personality could explain both child outcomes and household structure. Similarly, health challenges of either parents or grandparents could contribute to children’s care quality and the choice to coreside in a three-generational household. Yet this bias is unlikely to be resolved empirically due to ethical concerns with randomization and the difficulty of finding suitable instrumental variables; the approaches used are appropriate strategies given the context, restricting conclusions about the ability to infer the underlying mechanism.

This study has a number of strengths. Most importantly, the high number of household transitions experienced by children in Peru between ages 5 and 15 provides sufficient variation for within-child comparisons of child development outcomes for a variety of non–nuclear family types. Additionally, the cognitive tests are direct observations. Multiple analyses considering direction, timing, and child fixed effects result in comprehensive insights.

Though findings suggest that grandparent coresidence at the earliest ages may hinder cognition, policy should not discourage coresidence but rather provide additional support to both children and grandparents. For example, trainings and curriculum of the home-visiting program Cuna Mas could be expanded intentionally to include coresident grandparents who are not primary caregivers. Though there are reports of some success in engaging fathers and additional family members, the focus – and time burden – has been on mothers (Boyd and Rentería 2018). Instructing grandparents with lower levels of education on how to best interact with children to promote early cognitive development (for example, with play, singing, and talking) might help improve child outcomes. Different policies are needed for school-age children, for whom changes in father coresidence are associated with math scores. Of note, there is no gender gap in math scores found among 19-year-olds in the older Young Lives cohort (Singh and Krutikova 2017), and in Latin America, girls have consistently higher academic performance than boys (Grant and Behrman 2010). Thus mothers likely have the same mathematical competency as fathers. However, they may not interact with children in the same way fathers do; more research is needed to explore this hypothesis. If this is true, school-facilitated parent workshops could encourage mothers (and grandparents) to broaden their interactions within the mathematical realm. Additionally, strengthening fatherhood programs also can help, though engaging fathers is generally difficult, particularly when they are not coresident. It is even more difficult in a population with high rates of migration (Aguayo, Barker, and Kimelman 2016).

In any case, this research indicates that policymakers need to consider family in childhood to be a diverse and even fluid concept. The stable nuclear family is experienced by a minority of Peruvian children. The process of creating social policy to interact with education policy must incorporate a variety of family contexts and consider the transitions that children experience. Particularly important is supporting the academic processes of children with absent fathers. Hopefully these policies will result in more effective and equitable interventions for children in non-nuclear families.

## Supplementary Material

read-me

cleaning & analysis do files

## Figures and Tables

**Table 1:** Summary statistics (mean and standard deviation) by transition type

		All	No transition	Any transition	Father separation	Father union	Grandparent separation	Grandparent union
Child variables	Vocabulary – age 15 (standardized)	0.02	0.00	0.03	0.01	0.15	0.11	0.14
		(0.02)	(0.03)	(0.03)	(0.05)	(0.09)	(0.05)	(0.06)
	Math – age 15 (standardized)	0.00	−0.01	0.01	−0.08	−0.03	0.01	0.03
		(0.02)	(0.03)	(0.03)	(0.06)	(0.09)	(0.05)	(0.06)
	On-grade in school at age 15	0.81	0.81	0.81	0.74	0.76	0.82	0.81
		(0.01)	(0.01)	(0.01)	(0.02)	(0.04)	(0.02)	(0.02)
	Female	0.50	0.51	0.49	0.48	0.53	0.47	0.50
		(0.01)	(0.02)	(0.01)	(0.03)	(0.04)	(0.03)	(0.03)
	Firstborn	0.43	0.39	0.45	0.42	0.55	0.62	0.58
		(0.01)	(0.02)	(0.01)	(0.03)	(0.04)	(0.02)	(0.03)

Mother variables	Mother’s years of schooling	26.81	27.16	26.55	26.33	25.20	24.47	24.97
		(0.15)	(0.22)	(0.21)	(0.35)	(0.55)	(0.30)	(0.38)
	Mother’s native language indigenous	7.52	7.39	7.62	7.55	7.86	8.10	8.13
		(0.09)	(0.14)	(0.12)	(0.22)	(0.36)	(0.20)	(0.25)

SES variables	Housing quality index	0.34	0.32	0.35	0.31	0.28	0.26	0.26
		(0.01)	(0.02)	(0.01)	(0.02)	(0.04)	(0.02)	(0.03)
	Access to services index	0.47	0.46	0.48	0.48	0.49	0.49	0.50
		(0.00)	(0.01)	(0.01)	(0.01)	(0.02)	(0.01)	(0.01)
	Consumer durables index	0.38	0.38	0.39	0.38	0.39	0.39	0.40
		(0.01)	(0.01)	(0.01)	(0.01)	(0.02)	(0.01)	(0.02)

Geographic variables – proportions	Rural	0.68	0.65	0.69	0.70	0.70	0.69	0.73
	Region – coast	0.35	0.33	0.36	0.37	0.37	0.38	0.38
	Region – jungle	0.31	0.34	0.28	0.28	0.27	0.29	0.25
	Region – sierra	0.14	0.13	0.15	0.16	0.15	0.13	0.13
	
	N	2052	886	1166	351	136	391	255
	Proportion	1.00	0.43	0.57	0.18	0.07	0.20	0.13

*Source*: Young Lives Peru.

*Notes*: All variables from age 5 included except outcome variables. Any transition includes changes between household structure categories as described in the methods section. *Father separation, father union*, and *grandparent separation* are not mutually exclusive categories.

**Table 2:** Value-added models testing instability

	Vocab (standardized)		Math (standardized)		On-grade	
	No controls	Controls	No controls	Controls	No controls	Controls
Number of transitions*	0.01	0.01	0.02	0.01	−0.02	−0.03
	(0.02)	(0.02)	(0.03)	(0.03)	(0.01)	(0.01)
Number of moves (changed community)	0.07	0.07	0.05	0.03	0.01	0.01
	(0.02)	(0.02)	(0.03)	(0.02)	(0.01)	(0.01)
Outcome at age 5	0.59	0.47	0.36	0.24		
	(0.02)	(0.03)	(0.03)	(0.03)		

Adjusted R2	0.303	0.343	0.068	0.208	0.001	0.101
N	1499	1494	1740	1735	1747	1742

*Source*: Young Lives Peru, focus children survey rounds 1–5.

*Notes:* Controls are females, firstborn, attends school, mother and father education, mother’s indigenous status, mother’s age, wealth indices (housing quality, access to services, consumer durables), and geographic variables (rural and dummies for coast, jungle, and mountain regions).

* Between measurements at age 5 and age 15. Maximum number of transitions is three.

**Table 3:** Value-added models testing direction of transition

	Vocab (standardized)		Math (standardized)		On-grade	
	No controls	Controls	No controls	Controls	No controls	Controls
Father separation (experienced by 16%)	−0.12	−0.12	−0.14	−0.12	−0.11	−0.11
	(0.06)	(0.06)	(0.07)	(0.06)	(0.03)	(0.03)
Adjusted R2	0.294	0.337	0.071	0.215	0.011	0.109
N	1160	1160	1369	1369	1374	1374

Father union (experienced by 10%)	−0.13	−0.11	0.19	0.04	0.07	0.06
	(0.13)	(0.13)	(0.25)	(0.23)	(0.07)	(0.07)
Adjusted R2	0.447	0.502	0.03	0.199	−0.003	0.083
N	278	273	309	304	315	310

Grandparent separation (experienced by 51%)	0.1	0.09	0.02	0.11	0.02	0.05
	(0.08)	(0.08)	(0.10)	(0.09)	(0.04)	(0.04)
Adjusted R2	0.343	0.425	0.062	0.261	0.002	0.101
N	319	316	360	357	361	358

Grandparent union (experienced by 6%)	−0.02	−0.04	−0.03	−0.14	0.03	−0.01
	(0.09)	(0.15)	(0.13)	(0.04)	(0.04)
Adjusted R2	0.358	0.396	0.06	0.234	0	0.093
N	1078	1076	1254	1252	1270	1268

*Source*: Young Lives Peru, focus children in all survey rounds 1–5.

*Notes*: Transition occurs between ages 5 and 15. All regressions control for presence of the other family member (father/grandparent) in the household at age 5 and the number of moves between ages 5 and 15. Controls are female, firstborn, mother and father education, mother’s indigenous status, mother’s age wealth indices (housing quality, access to services, consumer durables), and geographic variables (rural and dummies for coast, jungle, and mountain regions). Vocab and math regressions control for standardized scores at age 5. Exit sample sizes were determined by number of households with that family member at age 5. Entrance sample sizes were determined by number of households without that family member at age 5. Percentages were determined from the on-grade sample.

**Table 4:** Child fixed effects

Base case is nuclear family	TVIP (standardized)				Math (standardized)		

	No controls	Controls	Lag* + controls	Includes siblings	No controls	Controls	Lag* + controls
Both parents + grandparent(s)	0	−0.01	0.02	0.02	−0.03	−0.03	−0.12
	(0.04)	(0.04)	(0.04)	(0.04)	(0.07)	(0.07)	(0.06)
Mother + grandparent(s)	−0.09	−0.1	−0.01	−0.08	−0.15	−0.15	−0.09
	(0.06)	(0.06)	(0.05)	(0.06)	(0.09)	(0.09)	(0.08)
Single mother	−0.02	−0.02	−0.01	−0.01	−0.15	−0.13	0.05
	(0.05)	(0.05)	(0.05)	(0.05)	(0.07)	(0.07)	(0.07)

Adjusted R2	0.682	0.686	0.685	0.683	0.474	0.474	0.473
N (observations)	7062	7053	6988	8941	7559	7550	7483
Number of children	1980	1980	1963	2780	1998	1998	1983

*Source*: Young Lives Peru, focus children in survey rounds 1–5.

*Notes*: All regressions control for the *stepfather, both parents* + *other adult*, and *no mother* family types. They also control for survey round indicators, child age, and their interactions. Additional controls are housing quality, services, and consumer durable indices, if the child attends school, if the child moved to a different community since last surveyed, number of children age 0–6 in the household, number of boys age 7–15 in the household, and number of girls age 7–15 in the household. Standard errors are clustered by child or, in the case of the included siblings, family.

* The lagged model uses the family structure from the previous round.

**Table 5:** Heterogeneity analysis for father separation using value-added models

	More advantaged	Vocab	Math	On-grade	Less advantaged	Vocab	Math	On-grade
**Panel A**	Urban	−0.13	−0.06	−0.08	Rural	−0.09	−0.29	−0.21
		(0.06)	(0.08)	(0.03)		(0.15)	(0.11)	(0.06)
	
	Adjusted R2	0.292	0.138	0.079	Adjusted R2	0.301	0.218	0.081
	N	910	941	943	N	250	428	431

**Panel B**	Father’s education secondary or more	−0.07	−0.12	−0.11	Father’s education primary or less	−0.29	−0.14	−0.12
		(0.07)	(0.08)	(0.03)		(0.12)	(0.10)	(0.06)
	
	Adjusted R2	0.261	0.129	0.068	Adjusted R2	0.277	0.134	0.052
	N	846	903	905	N	314	466	469

**Panel C**	Grandparent education secondary or more	0.05	0.13	−0.05	Grandparent education primary or less	−0.27	−0.18	−0.22
		(0.12)	(0.24)	(0.06)		(0.12)	(0.13)	(0.06)
	
	Adjusted R2	0.58	0.065	0.221	Adjusted R2	0.421	0.342	0.106
	N	98	101	101	N	197	229	230

*Source*: Young Lives Peru, focus children in all survey rounds 1–5.

*Notes*: Transition occurs between ages 5 and 15. Vocabulary and math regressions control for standardized scores at age 5. Controls are grandparent in household at age 5, number of moves between age 5 and 15, female, firstborn, mother and father education, mother’s indigenous status, mother’s age, wealth indices (housing quality, access to services, consumer durables), and geographic variables (rural and dummies for coast, jungle, and mountain regions). Sample sizes were determined by number of households with father present at age 5.

**Table 6:** Child fixed effects stratification analyses

	More advantaged				More disadvantaged			
			Vocab	Math			Vocab	Math
**Panel A**	Urban	Both parents + grandparent(s)	−0.03	−0.12	Rural	Both parents + grandparent(s)	0.09	0.23
			(0.05)	(0.07)			(0.10)	(0.13)
		Mother + grandparent(s)	– 0.08	– 0.02		Mother + grandparent(s)	– 0.12	– 0.6
			(0.07)	(0.10)			(0.15)	(0.21)
		Single mother	−0.05	−0.07		Single mother	0.12	−0.32
			(0.06)	(0.08)			(0.12)	(0.15)

		Adjusted R2	0.647	0.45		Adjusted R2	0.599	0.445
		N (observations)	5081	5160		N (observations)	1972	2390
		N (children)	1359	1365		N (children)	621	633

**Panel B**	Father’s education secondary or more	Both parents + grandparent(s)	0	−0.09	Father’s education primary or less	Both parents + grandparent(s)	0	0.15
			(0.05)	(0.07)			(0.09)	(0.14)
		Mother + grandparent(s)	−0.06	−0.14		Mother + grandparent(s)	−0.21	−0.27
			(0.07)	(0.10)			(0.17)	(0.22)
		Single mother	−0.02	−0.16		Single mother	0	−0.12
			(0.06)	(0.08)			(0.11)	(0.13)

		Adjusted R2	0.651	0.453		Adjusted R2	0.607	0.433
		N (observations)	4919	5083		N (observations)	2134	2467
		N (children)	1341	1347		N (children)	639	651

**Panel C**	Grandparent education secondary or more	Both parents + grandparent(s)	0	0.24	Grandparent education primary or less	Both parents + grandparent(s)	−0.04	−0.22
			(0.08)	(0.16)			(0.07)	(0.10)
		Mother + grandparent(s)	0.02	0.24		Mother + grandparent(s)	−0.17	−0.31
			(0.12)	(0.18)			(0.11)	(0.15)
		Single mother	0.06	0.21		Single mother	−0.05	−0.23
			(0.10)	(0.29)			(0.11)	(0.15)

		Adjusted R2	0.698	0.476		Adjusted R2	0.682	0.475
		N (observations)	682	685		N (observations)	1546	1652
		N (children)	183	183		N (children)	430	434

*Source*: Young Lives Peru, focus children in survey rounds 2–5.

*Notes*: All regressions control for survey round indicators, child age, and their interactions. Additional controls are housing quality, services, and consumer durable indices, if the child attends school, if the child moved to a different community since last surveyed, number of children age 0–6 in the household, number of boys age 7–15 in the household, and number of girls age 7–15 in the household. Standard errors are clustered by child.

## Data Availability

The data are publicly available from Young Lives: https://www.younglives.org.uk/content/use-our-data.

## Footnotes

2 Reading comprehension continues to develop during this time, though it does eventually plateau (Francis et al. 1996).

3 Likely the trend emerged because the most rural and thus most disadvantaged were more difficult to find for follow-up. Thus lower scores were associated with older ages.

4 Because vocabulary was standardized continuously over age instead of standardized separately by rounds (as for math), some age-dependence was found when controlling for the round of data collection. This could also be due to differences in knowledge of the focus children and younger siblings.

5 Though the age-standardized outcome variables are centered at 0, the standardization also includes observations from siblings, while the regression analysis includes only focus children. These survey round indicator variables and their interactions with age adjust for this difference in population.

6 The next younger sibling was tested if he or she was age 5 or older, starting when the focus child was age 8.

## Appendix: Justification for pooling grandparent types

To determine which family structure types to compare, I used categories defined by any or no grandparents. For example, a single mother living with her child’s maternal grandmother and one living with her child’s paternal grandfather would both be included in the *mother* + *grandparent* family category. To arrive at this delineation, families with grandmothers were compared to families with grandfathers, and families with maternal grandparents were compared to families with paternal grandparents. Families with different grandparent types were also taken into consideration. For example, there are only four families in which a single mother lives with paternal grandparents. Table A-3 shows the portion of children living with grandparents of different types, with the maternal grandmother having the highest likelihood of being in the houshold for all rounds.

The child fixed effects analysis was used, but different family structure variables were substituted in. Dummy variables for biological parents (both, mother only, father only, neither) and dummy variables for grandparents were included. The categorization “none, grandmother only, grandfather only, both” was tested and also the categorization “none, maternal grandparent(s) only, paternal grandparent(s) only, both.” For either categorization of the grandparents, no significant differences between grandparent types’ association with child development scores were found (Table A-4). However, when the categorical variable for biological parents in the household was interacted with the categorical varaible for grandparents in the household, a few categories in which there were significant differences were found (not shown). The interaction with the most signifiance was both paternal and maternal grandparents with a single mother. However, since there is only one child with this family structure, the grandparent types were pooled.

**Table A-1:** Portion of children living with this type of relative, by family type

	Nuclear	Both parents + grand(s)	Mother + grand(s)	Single mother	Stepfather	Both parents + other adult	No mother
Biological mother	1	1	1	1	1	1	0
Biological father	1	1	0	0	0	1	0.36
Stepmother	0	0	0	0	0	0	0.12
Stepfather	0	0	0	0	1	0	0.02
Grandparent	0	1	1	0	0.13	0	0.56
Uncle/aunt	0	0.44	0.65	0.11	0.08	0.43	0.46
Other	0	0.05	0.04	0.07	0.06	0.61	0.19

N	5709	1027	700	808	381	533	560
Percent of observations	59%	11%	7%	8%	4%	5%	6%

*Source*: Young Lives Peru, focus children in rounds 1–5, all observations.

*Note*: “Other” includes nephews, nieces, or other relatives.

**Table A-2:** Sample size

	5 years	8 years	12 years	15 years	All rounds	Any round
Vocab (focus child)	1686	1712	1850	1816	1444	1980
Vocab (sibling)		438	733	739	388	802
Math	1949	1881	1871	1860	1737	1998

*Source*: Young Lives Peru.

**Table A-3:** Portion of children living with different grandparents in each survey round

Focus children	Age 1	Age 5	Age 8	Age 12	Age 15	Ever	% with transition*
Any grandparent	31.7%	23.3%	19.8%	17.2%	16.2%	42.2%	35.0%
Maternal grandmother	25.3%	16.1%	13.6%	11.5%	10.7%	32.8%	28.1%
Maternal grandfather	18.9%	10.8%	8.3%	7.5%	6.8%	23.1%	19.8%
Paternal grandmother	0.0%	5.0%	4.4%	3.5%	3.1%	7.8%	7.8%
Paternal grandfather	0.0%	3.4%	3.1%	2.2%	1.9%	5.2%	5.2%

N = 1,813							

*Source*: Peru Young Lives, focus children in all rounds.

*Notes*: Age 1 questionnaire does not distinguish between maternal and paternal grandparents. Estimates were based on identity of grandparents at child age 5. Unclassifiable grandparents (those no longer in the household in R2) were assumed to be maternal.

* A change in presence of the family member in the household roster.

**Table A-4:** Child fixed effects regressions contrasting grandparent classification

		Vocab (standardized)		Math (standardized)	
Panel A	Child fixed effects	No controls	Controls	No controls	Controls
Grandparent indicator variables – base category is no grandparents	Grandmother	0.01	0.04	0	−0.13
		(0.05)	(0.04)	(0.07)	(0.07)
	Grandfather	0.01	0	0.03	−0.07
		(0.07)	(0.07)	(0.13)	(0.12)
	Both	−0.06	−0.03	−0.05	−0.09
		(0.05)	(0.04)	(0.07)	(0.06)
Parent indicator categories – base category is both	Mother only	−0.03	−0.03	−0.13	−0.11
		(0.05)	(0.05)	(0.06)	(0.06)
	Father only	0.01	0	−0.05	−0.03
		(0.07)	(0.07)	(0.11)	(0.11)
	Neither	0.09	0.05	−0.13	−0.13
		(0.08)	(0.08)	(0.10)	(0.10)

	Adjusted R2	0.682	0.685	0.473	0.473
	N (observations)	7064	6989	7561	7484
	N (focus children)	1980	1963	1998	1983
	P value*	0.96	0.60	0.85	0.65
		Vocab (standardized)		Math (standardized)	
Panel B	Child fixed effects	No controls	Controls	No controls	Controls

Grandparent indicator variables – base category is no grandparents	Paternal grandparent(s)	−0.10x	0.01	−0.1	−0.05
		(0.06)	(0.06)	(0.09)	(0.09)
	Maternal grandparent(s)	0	0.01	0.02	−0.10x
		(0.04)	(0.03)	(0.06)	(0.05)
	Both	−0.09	0.26	−0.22	0.39
		(0.18)	(0.18)	(0.25)	(0.35)
Parent indicator categories – base category is both	Mother only	−0.03	−0.03	−0.13xx	−0.11x
		(0.05)	(0.05)	(0.06)	(0.06)
	Father only	0.02	0	−0.04	−0.03
		(0.07)	(0.07)	(0.11)	(0.11)
	Neither	0.08	0.05	−0.14	−0.13
		(0.08)	(0.08)	(0.10)	(0.10)

	Adjusted R2	0.682	0.685	0.474	0.473
	N (observations)	7064	6989	7561	7484
	N (focus children)	1980	1963	1998	1983
	P value*	0.14	0.99	0.27	0.66

*Note*: Controls are as in Table 3.

* P value is the test of equality of coefficients on grandmother and grandfather.

*Notes*: Controls are as in Table 3.

* P value is the test of equality of coefficients on paternal and maternal grandparents.
